# Puzzle Hi-C: An accurate scaffolding software

**DOI:** 10.1371/journal.pone.0298564

**Published:** 2024-07-15

**Authors:** Guoliang Lin, Zhiru Huang, Tingsong Yue, Jing Chai, Yan Li, Huimin Yang, Wanting Qin, Guobing Yang, Robert W. Murphy, Ya-ping Zhang, Zijie Zhang, Wei Zhou, Jing Luo

**Affiliations:** 1 State Key Laboratory for Conservation and Utilization of Bio-resource, School of Ecology and Environment, School of Life Sciences and School of Medicine, Yunnan University, Kunming, Yunnan, China; 2 Department of Biology, University of Waterloo, Waterloo, ON, Canada; 3 Reptilia Zoo and Education Centre, Vaughan, ON, Canada; 4 Southwest United Graduate School, Yunnan University, Kunming, Yunnan, China; 5 National Pilot School of Software, Yunnan University, Kunming, Yunnan, China; University of Veterinary Medicine Vienna: Veterinarmedizinische Universitat Wien, AUSTRIA

## Abstract

High-quality, chromosome-scale genomes are essential for genomic analyses. Analyses, including 3D genomics, epigenetics, and comparative genomics rely on a high-quality genome assembly, which is often accomplished with the assistance of Hi-C data. Curation of genomes reveal that current Hi-C-assisted scaffolding algorithms either generate ordering and orientation errors or fail to assemble high-quality chromosome-level scaffolds. Here, we offer the software Puzzle Hi-C, which uses Hi-C reads to accurately assign contigs or scaffolds to chromosomes. Puzzle Hi-C uses the triangle region instead of the square region to count interactions in a Hi-C heatmap. This strategy dramatically diminishes scaffolding interference caused by long-range interactions. This software also introduces a dynamic, triangle window strategy during assembly. Initially small, the window expands with interactions to produce more effective clustering. Puzzle Hi-C outperforms available scaffolding tools.

## Introduction

Analysis of genomic data rely on high-quality chromosome-level genomes. Accuracy is essential for downstream genomic analyses, and especially for 3D, comparative, and functional genomic analyses. For example, due to significant interactions being calculated on linear distances of a draft genome, incorrect assembly leads to false positive interactions, resulting in unreliable 3D genome analysis results [1, 2]. Chromosome evolution and the estimation of recombination rely on the contiguity of the genome [3, 4]. Only chromosome-scale genomes can reveal the complexity of regulatory architecture and how cis-regulatory elements influence genes because some cis-regulatory elements are likely more than 1 Mb from the target gene [5–7]. A contiguous genome can significantly improve the interpretation of genome-wide association studies (GWAS) [8–11] because regions of linkage usually exist on the same contig. Thus, high-quality chromosome-level genomes are requisite for multiple downstream genomic analyses [12].

Long-read sequencing technologies have facilitated genome assembly because they yield large, overlapping repetitive regions. Notwithstanding, such contigs do not always stretch into a complete chromosome or even one arm of a chromosome. To obtain chromosome-scale scaffolds, various strategies have been explored to increase the contiguity of *de novo* genome assemblies. Two primary methods order and orient scaffolds for chromosome-level assembly: genetic mapping and high-throughput chromatin conformation capture (Hi-C). Traditional genetic mapping orders and orients scaffolds based on linkage groups. However, genetic maps require a large number of individual offspring to be sequenced. This dramatically limits the application of genetic maps in the genome because the sequencing of a large number of individuals costs time, computing resources, storage resources, and other expenses [13–16]. By contrast, recently developed Hi-C provides a powerful tool for chromosome-level assembly because it requires a small number of tissue samples to mount to scaffold contigs into chromosomes [17, 18]. Consequently, Hi-C is the most commonly used method for scaffolding at the chromosome level.

Three invariant features of Hi-C interactions are used for genome assembly [19]: intra-chromosomal interaction enrichment, the random positioning of chromosomes in the nucleus, and the local smoothness of interactions reflected in the Hi-C heat map. In the Hi-C interaction matrix, a locus tends to interact more frequently with another locus within the same chromosome (cis-interactions) than with loci on a different chromosome (trans-interactions). Two phenomena of 3D chromatin may contribute to this feature. In the first phenomenon, chromosomes occupy distinct volumes throughout the cell cycle, leading to physical separation between chromosomes [20, 21]. The second phenomenon relies on the random positioning of chromosomes in the nucleus [22], which may largely reduce chromosomal interactions. The probability of intra-chromosomal interaction decreases with increasing linear distance. Thus, in Hi-C interaction maps, the frequency of interaction tends to decrease with genomic distance, i.e., a locus interacts more frequently with other loci that are nearby in the genomic space than with distant loci. When the distance is greater than 100kb, the probability of interaction is about 1*/x*, where *x* is the distance between two points [17, 23]. Finally, local smoothness of interactions as reflected in the Hi-C heat map interaction of adjacent points tends to be consistent [24]. Available software commonly use features one and two for scaffolding and the preferable software is the one that produces the fewest errors as revealed via genome curation.

Accompanying the sharply decreasing price of genomic sequencing, Hi-C data for scaffolding at chromosome-level is now accessible and popular. The first Hi-C scaffolding software, LACHESIS [17], developed in 2013, has seen increasingly employment for constructing chromosome-level in genomes. Although several software options exist for Hi-C scaffolding [17, 25–28], none can eliminate errors, including artificial relocations, translocations, and inversions [26, 28], that result in false assemblies and erroneous genomes. Chromosomes may fold into various structures, such as loops, topological associated domains (TADs), and compartments, which lead to many long-range interactions. Such interactions violate the assumption that the probability of the intra-chromosomal interaction decreases with the linear distance, thus causing an incorrect assembly. Some methods use a contig-end solution [29, 30], but the employment of limited information results in disappointing performance. To address these issues, we offer an easy-to-use Hi-C scaffolding software, Puzzle Hi-C, which uses a triangle window and iterative assembly strategy to reduce long-range interactions thereby improving performance. This software achieves outstanding scaffolding results in both simulated data and real data. The source code and documentation of Puzzle Hi-C are available at GitHub (https://github.com/linguoliang/puzzle-hi-c.git).

## Methods

### Datasets

We used human Hi-C data from the GM12878 cell line and thale cress (*Arabidopsis thaliana*) as benchmark data. Exemplar genomes used Hi-C data from a gayal (*Bos frontalis*) and puffer fish (*Takifugu bimaculatus*). We also used the Hi-C data from a broomcorn millet (*Panicum miliaceum*), Indian cobra (*Naja naja*), Peking duck (*Anas platyrhynchos*), water buffalo (*Bubalus bubalis*), yellow croaker (*Larimichthys crocea*), fighting fish (*Betta splendens*) and flower thrips (*Frankliniella intonsa*) (S1 Table in S1 File).

### Puzzle Hi-C pipeline

**Fig 1:**
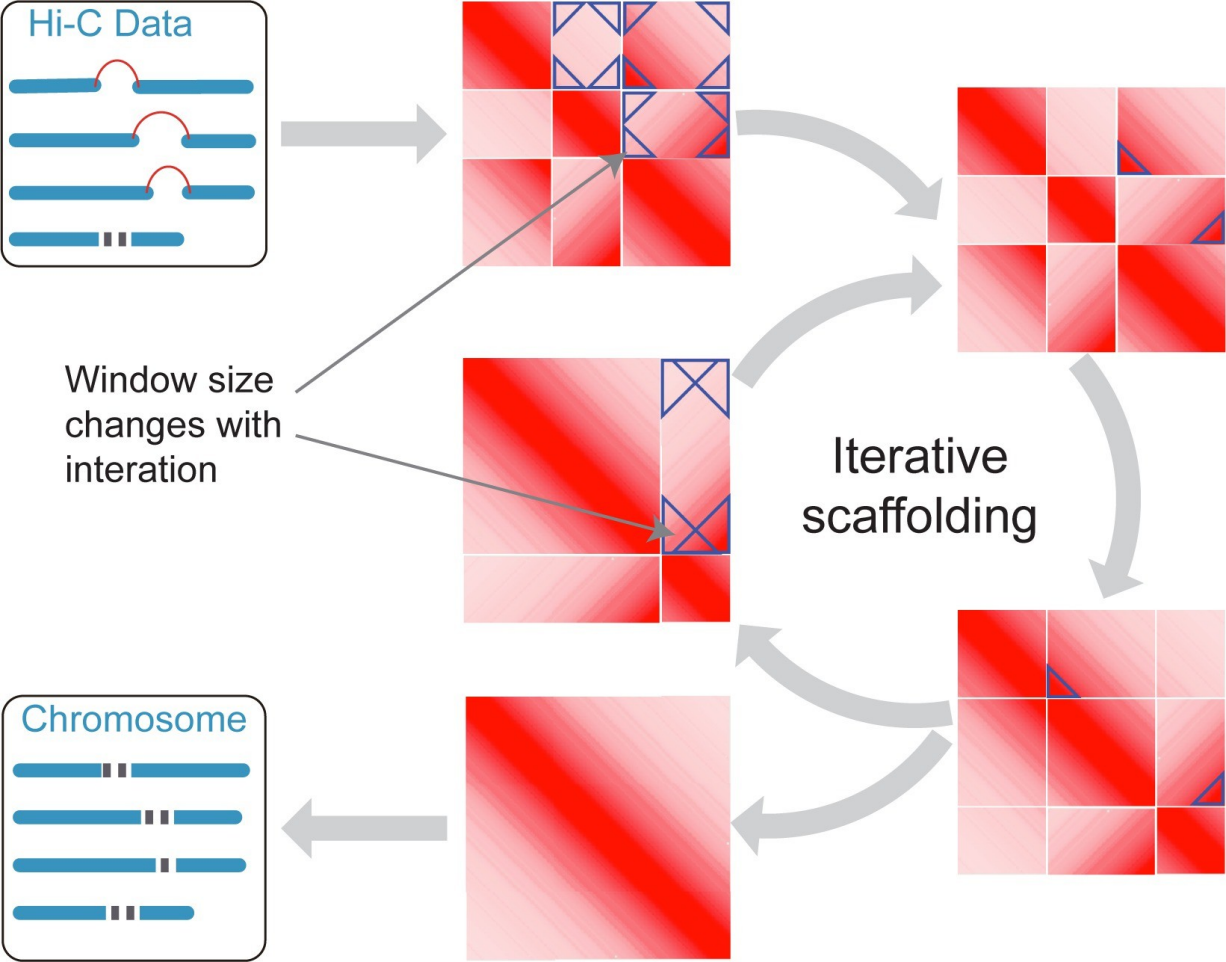
Puzzle Hi-C pipeline. The Puzzle Hi-C pipeline contains three steps: mapping, scaffolding, and building. Ordering and orientation adopt an iterative method to obtain accurate assemblies via multiple iterations. Puzzle Hi-C introduces a dynamic, triangle window strategy during assembling. The triangle window is initially small but expands with interactions to produce more effective clustering. Finally, the genome is assembled according to the scaffolding results and output in final fasta and apg format files.

The Puzzle Hi-C pipeline contains three steps: mapping, scaffolding, and building. Briefly, Puzzle Hi-C uses Juicer software for the first mapping step. Next, scaffolding inputs and iteratively merges contigs into chromosomes. Finally, building reconstructs each chromosome by concatenating the contigs, adding gaps between the contigs, and generating the genome in the FASTA format (Fig 1).

### Puzzle Hi-C mapping

Puzzle Hi-C uses Juicer software for mapping. Juicer uses bwa mem default parameters for mapping. After mapping, the Juicer program filters out PCR duplicates and only retains the result on one pairwise comparison.

### Puzzle Hi-C scaffolding

The iterative algorithm used for scaffolding solves two problems: "ordering" assigns a relative position to each scaffold on each chromosome with respect to the other scaffolds assigned to the same chromosome; and "orienting" determines which of the two ends of each scaffold is adjacent to the preceding scaffold, and which end is adjacent to the next scaffold. In each step, subsets of the input scaffolds are ordered and oriented with respect to one another to create a new, longer set of scaffolds, which are then used as inputs for the next step until all contigs are assigned to scaffolds that match the user-specified number of chromosomes. The software uses a weight matrix to build a graph and the graph nodes are the scaffolds in a chromosome-group. Weight is defined as follows:

Each end of each scaffold is labeled using B (begin) and E (end). Given two scaffolds *i* and *j*, there are four possible connections, BB, BE, EB, and EE. We defined a length cutoff of *l* and considered the read pairs mapped in the region of length *l* at both ends (B and E) of the scaffolds.

The number of links was determined using:

$$N_{i,j}=\max\left\{N_{iB,jB},N_{iB,jE},N_{iE,jB},N_{iE,jE}\right\}$$

For each scaffold *i*, we only considered the top 5 linked edges. Next, we obtained a link-score for each pair as follows:

$$W_{topk}=\frac{N_{topk}}{\sum_{j=1}^{5}N_{topj}}$$

When ordering the scaffolds, and because each node could only have two edges, we retained the two edges with the largest and second-largest weight. We set a cutoff for the weight; if the weight was less than the cutoff, then the connection was considered unreliable and removed. The graph found the path with all nodes > 1. Subsequently, the software constructed new scaffolds according to the path and direction of connection for the next iteration. For each iteration, the length of *l* was increased with the growth rate 1.4 so that every two iterations doubled the length. The length of contigs or scaffolds less than *l* were then filtered out for scaffolding. The iteration stopped only if the number of scaffolds equaled to or were close to the specified number of chromosomes.

### Puzzle Hi-C building

Once scaffolding is completed, Puzzle Hi-C builds a chromosome-level genome assembly. Scaffolds link with 100 bp N gaps (N can be configured with Puzzle Hi-C parameters). Puzzle Hi-C also generates an agp file to record how scaffolds are assembled with the position and direction information.

### Genomic collinearity

The genomic collinearity analysis between genomes were completed using NUCMER from the MUMmer package v3.23 with default parameters [31]. After alignment, we sorted the scaffolds according to collinearity to draw the final collinearity figures.

### Generation of simulated data

We used chromosome-scale genomes from human and thale cress to generate small contig genomes by splitting genomes in 200 kb, 400 kb, 600 kb, 800kb or 1 Mb contigs. Other chromosome-scale genomes were split into scaffolds based on their existing 100 bp, 200 bp, 500 bp or 1 kb gaps.

### Scaffolding error statistics

To compare the performance of each software, we aligned the genome assembly to their respective reference genomes using the program nucmer with default parameters. Alignment quality was assessed using dnadiff [31], a MUMmer utility that provides detailed information on the differences between two genomes. We also employed Edison [32] software to calculate the minimum number of edits (splitting scaffolds, joining scaffolds, moving contigs, and inverting contigs) to make the assembled genome identical to reference genome, which was then referred to as edit distance. To get a reliable result, we sampled scaffolding 25 times, where each sample contained 5 chromosomes.

### Hi-C scaffolding in LACHESIS, SALSA2, 3D-DNA, ALLHiC, and YaHS

Hi-C reads were mapped using bwa with default parameters. The SAM file, which was generate by bwa, was filtered using PreprocessSAMs.pl.

For LACHESIS, we used default parameters except CLUSTER_N, which depended on how many chromosomes should be clustered.

For SALSA2, the minimum input files were provided with the following command line: python run_pipeline.py -a seq.fasta -l seq.fasta.fai -b alignment.bed -e GATC -o scaffolds.

For ALLHiC, we used the default parameters, except -k. The k parameter was set according to how many chromosomes were clustered.

For YaHS, we used the default parameters on a computer with 512 GB memory. Such was necessary because YaHS requires more than 370 GB memory to scaffold the human genome with a resolution of 10 kb.

For 3D-DNA, Hi-C reads were aligned by Juicer software using default parameters. Scaffolds >15 kb were retained, and the haploid model was selected in the 3D-DNA pipeline.

## Results

### Overview of the Puzzle Hi-C algorithm

**Fig 2:**
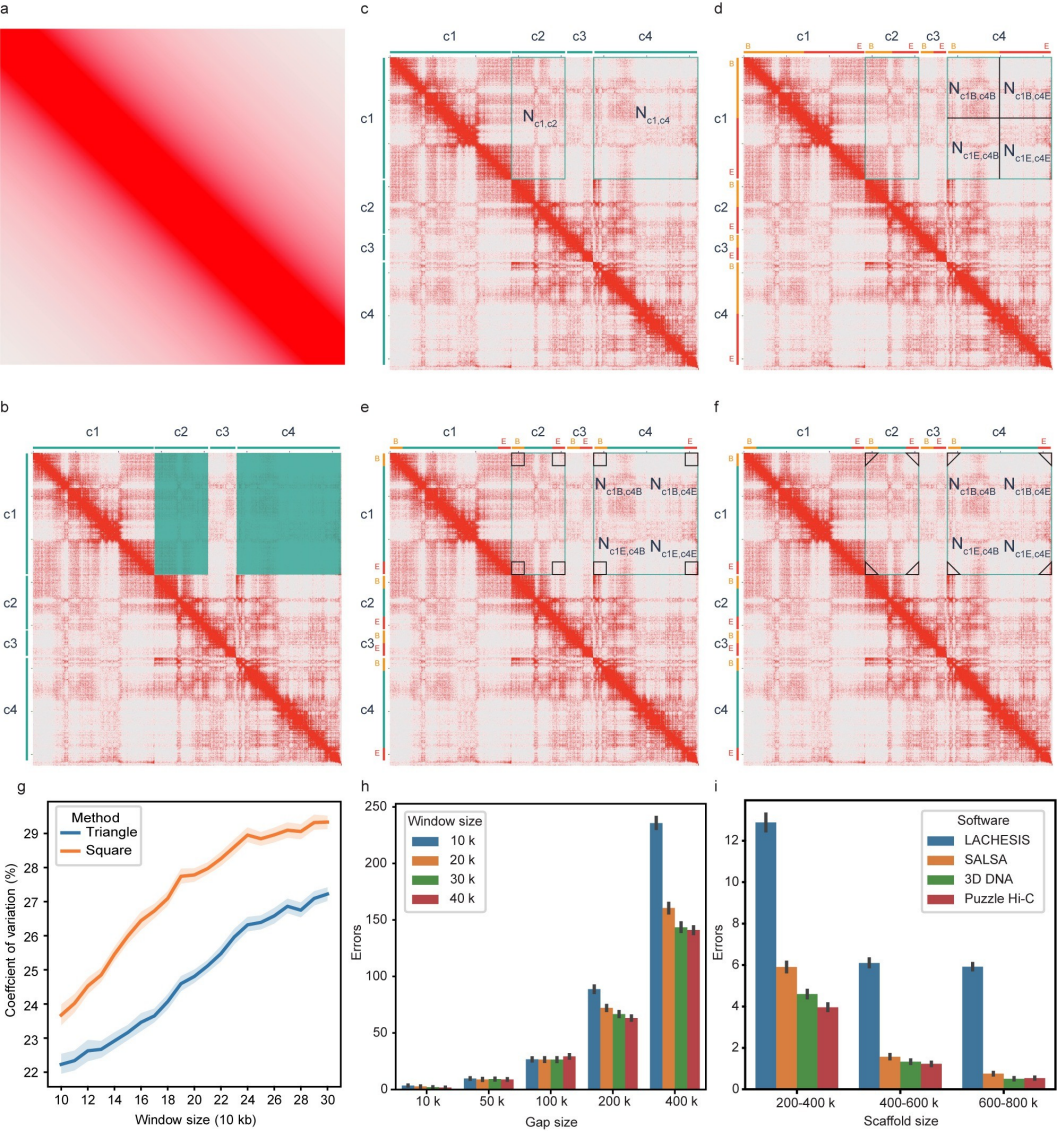
Contact probability and strategies used to evaluate distance between scaffolds adopted by different software. **a**, Ideally, the distribution of Hi-C contact is 1*/x*. Heat map shows the diagonal position of interaction density is very high, the farther away from the diagonal, the lower the interaction density becomes. **b**, Heat map shows the chr2 [0–35MB] assembled by LACHESIS, where c1, c2, c3 and c4 represent scaffolds and the rectangle represents the number of Hi-C reads links with two scaffolds. Compartment and TADs produce many long-range interactions with densities higher than adjacent interaction densities. **c**, Strategy to evaluate distance as adopted by LACHESIS and ALLHiC. **d**, Strategy to evaluate distance as adopted by 3D DNA. **e**, Strategy to evaluate distance as adopted by SALSA2. **f**, Strategy to evaluate distance as adopted by Puzzle Hi-C. **g**, CV of interaction density with the triangle region and square region. **h**, Errors of the distance between two scaffolds with different gap sizes in Puzzle Hi-C. **i**, Errors of different strategies to evaluate the distance between two scaffolds with 1000 samplings.

Comparative analysis of existing software shows that LACHESIS and ALLHiC use all information on interactions between scaffolds for clustering, and achieves high performance on clustering [17, 27]. In contrast, SALSA2 only uses partial information at both ends of the scaffold, which advances ordering and orientation [28]. Considering the accuracy of scaffold ordering and clustering, Puzzle Hi-C dynamically changes the size of the statistical window at both ends of the scaffold, and increases the window size as the number of iterations increases (Fig 1). Therefore, our software uses local information at both ends of the scaffold for ordering and orientation at the initial assembly. As the statistical window increases in size, Puzzle Hi-C uses global interactions for better clustering (Fig 2). Puzzle Hi-C contains three steps: mapping, scaffolding, and building. Mapping uses Juicer software [33] to filter out duplicate, abnormal alignments and restriction site information. Scaffolding adopts an iterative method to obtain accurate assemblies via multiple iterations. Finally, the genome is assembled into scaffolds according to the ordering and orientation results, and the genome is output in final fasta and apg format files.

### Evaluation using simulated and real data

To compare the performance of Puzzle Hi-C and other software, we used the human genome hg38. For the simulated data, we assessed the autosomes using lengths of 200 kb, 600 kb and 1 Mb contigs (S2-S7 Tables in S1 File). For example, the LACHESIS assembled genome size was 2.77 Gb and it yielded 102, 37, and 33 scaffolds, respectively. Scaffold N50s tended to be stable at about 135 Mb, while scaffold N90s were 79.8 Mb, 68.8 Mb, and 77.5 Mb, respectively (S2 Table in S1 File). SALSA2 assembled scaffolds of 1193 Mb, 593 Mb, and 538 Mb, respectively. Scaffold N50s were 8.6 Mb, 9.3 Mb, and 10.0 Mb, respectively (S3 Table in S1 File). The assembled scaffolds were relatively short and the clustering effect was not ideal, with scaffold N90s of only 1.2 Mb, 2.5 Mb, and 2.8 Mb, respectively. Except for 3D DNA and SALSA2, the other software obtained reasonable N50s and N90s.

**Fig 3:**
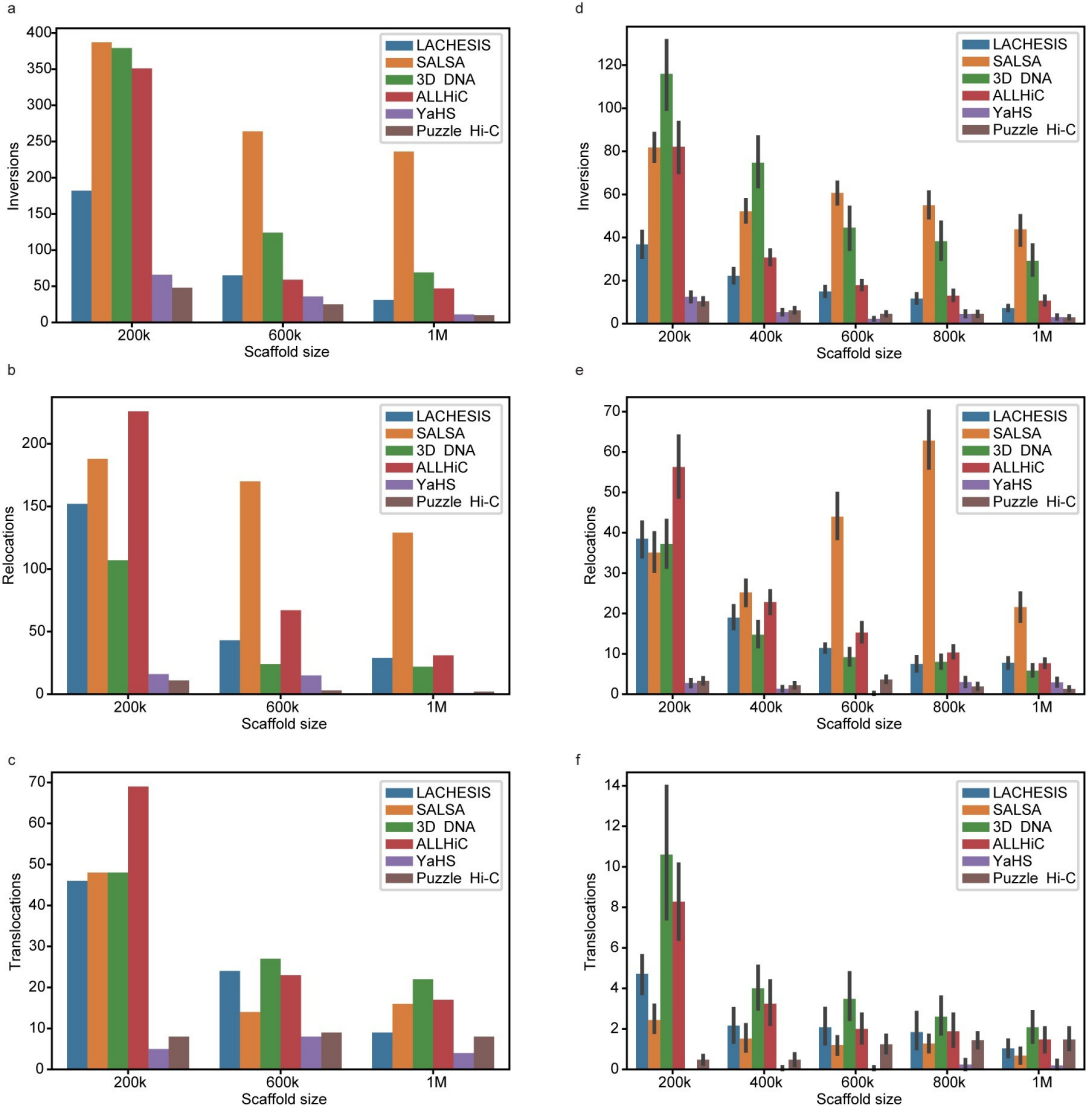
Number of different errors generated by different software with different scaffold size. **a-c,** inversions, relocations and translocations generated by LACHESIS, SALSA2, 3D DNA, ALLHiC, YaHS, and Puzzle Hi-C under different length of Scaffolds. **d-f,** Inversions, relocations and translocations generated by LACHESIS, SALSA2, 3D DNA, ALLHiC, YaHS, and Puzzle Hi-C with 25 sampling data under different length of scaffolds.

Second, to compare the performance of scaffolding software in scaffolding and orientation, we used dnadiff to assess three features: the number of relocations was determined by the number of breaks in the alignment of scaffolds belonging to the same chromosome, but not consistently ordered; the number of translocations, that being the number of breaks in the alignment of scaffolds belonging to different chromosomes; and the number of inversions, or breaks in the alignment by scaffolds inverted with respect to one another. We also used Edison to assess the edit distance. As the size of scaffolds became smaller, the proportion of assembly errors in LACHESIS increased (Table 1). It produced 69 assembly errors in the 1 Mb scaffold size, including 9 translocations, 31 orientation assembly errors, and 29 relocations; at the 600 kb scaffold size, assembly errors increased to 132, and the 200 kb size had 380 errors, which showed an inverse relationship between scaffold size and assembly errors. Other software showed the same pattern. The most widely used scaffolding program, YaHS, substantially outperformed all prior software. In 1 Mb scaffolds, it had one error in relocation, four in translocations and 11 inversions. Comparatively, Puzzle Hi-C usually achieved the greatest assembly accuracy under different sizes of scaffolds (Fig 3A–3C and Table 1), for example having 67 assembly errors at the 200 kb scaffold size (11 relocations, 8 translocations, 48 inversions), 37 errors at 600 kb, and 20 errors at 1 Mb. SALSA2 had the lowest edit distance, Puzzle Hi-C produced the second lowest edit distance except at 1 Mb.

**Table 1:** Number of errors generated by different software from human genome hg38 data split into different contig lengths.

Contig Length	Features	LACHESIS	SALSA2	3D-DNA	ALLHiC	YaHS	Puzzle Hi-C
**200 kb**	Relocations	152	188	107	226	16	11
	Translocations	46	48	48	69	5	8
	Inversions	182	387	379	351	66	48
	Edit distance	14250	13230	14055	14401	13795	13717
**600 kb**	Relocations	43	170	24	67	15	3
	Translocations	24	14	27	23	8	9
	Inversions	65	264	124	59	36	25
	Edit distance	5157	4641	5120	5217	5098	5047
**1 Mb**	Relocations	29	129	22	31	1	2
	Translocations	9	16	22	17	4	8
	Inversions	31	236	69	47	11	10
	Edit distance	3324	2904	3245	3373	3266	3286

We split genome hg38 into 200 kb, 600 kb and 1 Mb contig lengths, and then used these for scaffolding. Next, we used dnadiff to calculate errors (relocations, translocations and inversions) in scaffolding results compared to hg38, and we used Edison to calculate edit distances.

Third, we resampled the human genome 25 times with five different scaffold sizes. Puzzle Hi-C and YaHS outperformed the other software packages (Fig 3D–3F). Puzzle Hi-C was more robust as the errors decreased with increasing scaffold size and YaHS was more sensitive to scaffold size. YaHS had fewer errors with 600 kb scaffold size, but the error increased with scaffold size change (Fig 3D–3F and S8 Table in S1 File).

**Fig 4:**
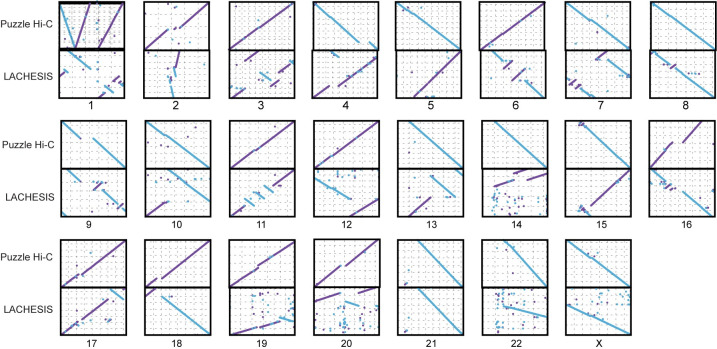
The synteny of chromosomes assembled by Puzzle Hi-C and LACHESIS compared with GRCh38. Synteny between the assembly of chromosomes from scaffolders and GRCh38 chromosomes.

To test the assembly performance of Puzzle Hi-C using real data, we also employed the scaffold version of the human genome assembly (version: GCA_001013985.1). This analysis compared LACHESIS and Puzzle Hi-C in detail, but also the other methods. We compared the assemblies to the human genome GRCh38 using MuMmer software. LACHESIS produced 999 errors in its ordering and orientation of large fragments in assembly, and Puzzle Hi-C gave 647 errors, except for chromosome 1, which was composed of three scaffolds. Although LACHESIS had problems in assembling small scaffolds, such as chromosomes 17, 19, 20, and 22, Puzzle Hi-C did not (Fig 4 and Table 2). All other methods produced more errors and edit distance than Puzzle Hi-C (Table 2). Puzzle Hi-C and YaHS produced similar results with the T2T genome of thale cress. However, YaHS was more likely to join the ends of telomeres together, thus producing more small scaffolds, and it exhibited difficulty in assembling the centromere (S1 Fig and S9 Table in S1 File).

**Table 2:** Number of errors generated by different software with human genome GCA_001013985.1.

Features	LACHESIS	SALSA2	3D-DNA	ALLHiC	YaHS	Puzzle Hi-C
**Relocations**	529	1076	4632	4188	527	243
**Translocations**	116	420	408	2526	250	149
**Inversions**	354	714	8572	2908	592	255
**Edit distance**	2992	5140	16480	18046	4305	2797

### Assembling genomes enriched with long-range interactions

**Fig 5:**
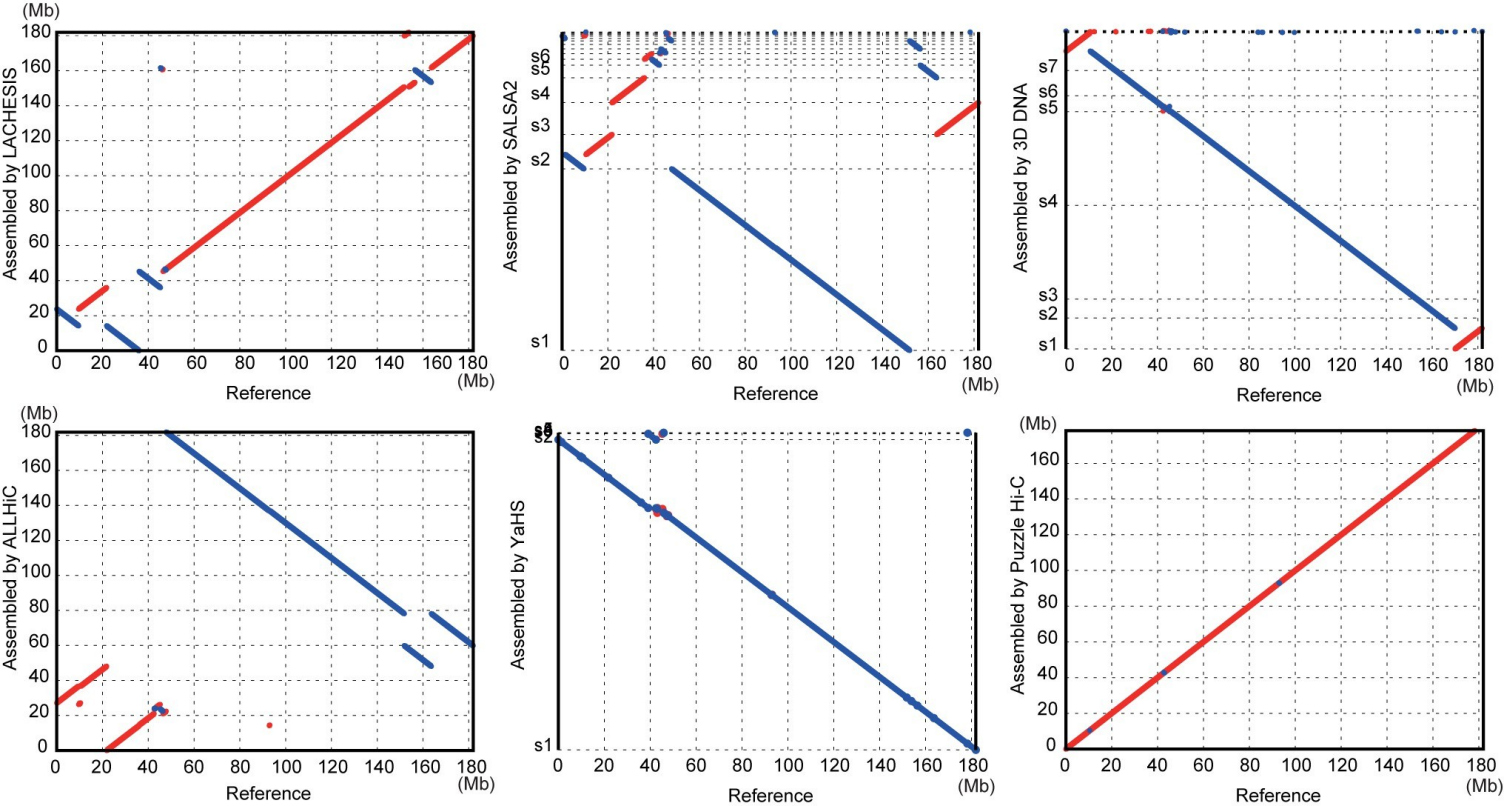
The synteny of chromosomes assembled by Puzzle Hi-C and other software compared with gayal chromosome 2. Synteny between the assembly of chromosomes from scaffolders (LACHESIS, SALSA2, 3D DNA, ALLHiC, YaHS and Puzzle Hi-C) and manually curated chromosome 2 of gayal.

To further test the robustness of assembly by Puzzle Hi-C, we employed chromosome 2 of gayal, which contains a Robertsonian translocation. This chromosome has more repetitive sequences and long-range interactions than its relatives. Long-range interactions obstruct ordering prediction. The Hi-C interaction matrix revealed a very strong internal interaction of compartments on chromosome 2, which indicated a long-distance interaction (S2 Fig in S1 File). Other software assemblies also detected the rearrangement of large fragments, but Puzzle Hi-C obtained relatively fewer chromatin orientation assembly errors. Therefore, Puzzle Hi-C appeared to best assemble chromosomes when chromatin interactions occurred (Fig 5). We also assembled other species genomes across a range of taxonomic groups, genome sizes and initial assembly quality. Puzzle Hi-C consistently generated assemblies with higher contiguity (S3 Fig in S1 File).

### The quality of genome is crucial for conducting 3D genome analysis

**Fig 6:**
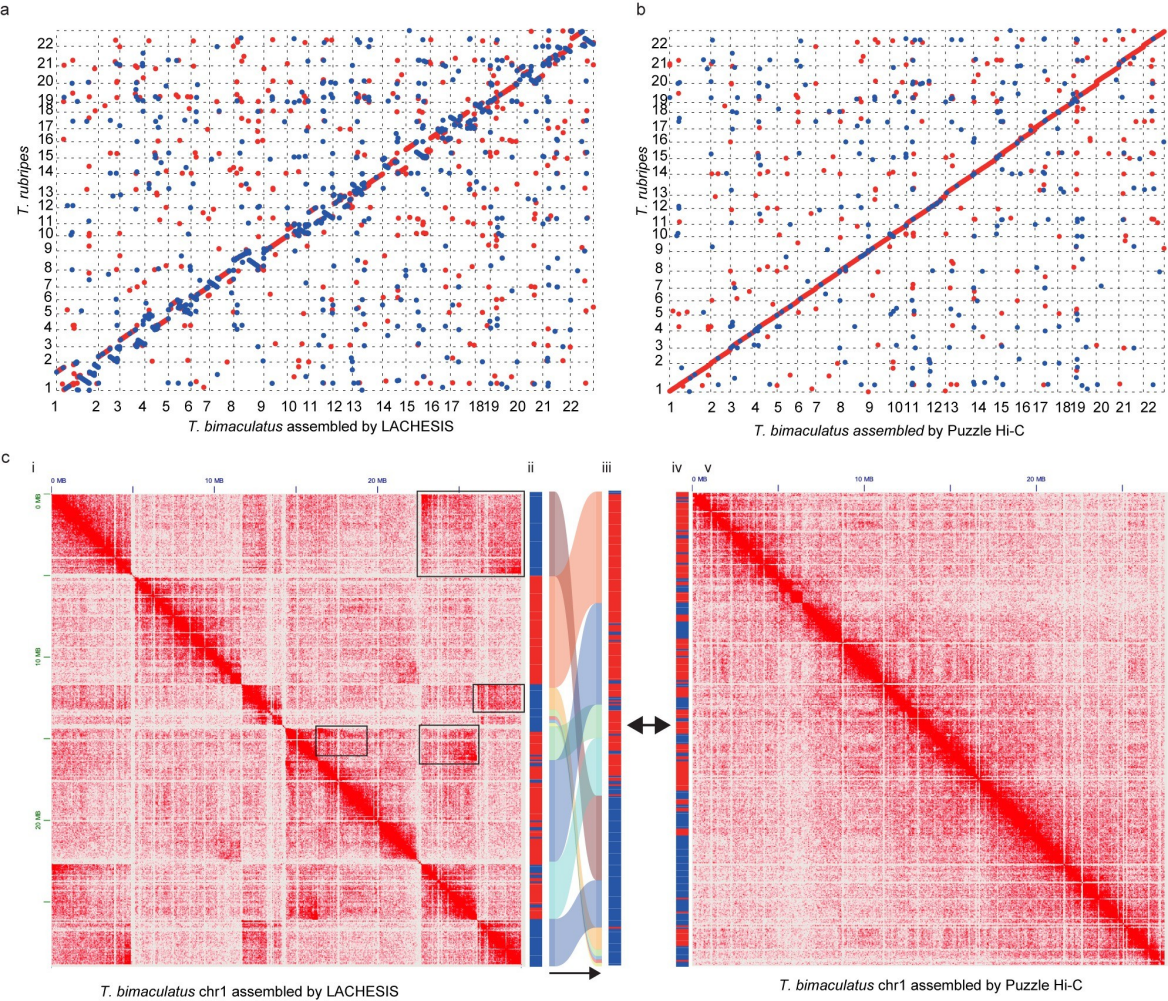
The scaffolding results of LACHESIS and Puzzle Hi-C on *T*. *bimaculatus*. **a**, Synteny between *T*. *bimaculatus* and *T*. *rubripes*; **b**, Synteny between Puzzle Hi-C-corrected *T*. *bimaculatus* and *T*. *rubripes*; **c**, i *T*. *bimaculatus* genome chr1 Hi-C heat map, the black box is the Hi-C heat map suggesting assembly error; ii *T*. *bimaculatus* genome chr1 Compartment, red is Compartment A and blue is Compartment B; iii The rearrangement of *T*. *bimaculatus* genome chr1 Compartment according to the corrected chr1; iv Puzzle Hi-C corrected chr1 Compartment after Puzzle Hi-C correction; v Puzzle Hi-C corrected chr1 Hi-C heat map.

A high-quality genome is essential for downstream analysis. However, some tools may produce some chromosome-level assembly errors that affect downstream analyses. To evaluate this, we downloaded the genome of *Takifugu bimaculatus* [34], which was assembled by LACHESIS. Compared to the genome of *T*. *rubripes*, the genome of *T*. *bimaculatus* had 809 inversions and 2618 relocations. We reassembled this genome using Puzzle Hi-C and obtained 519 inversions and 1791 relocations. We performed comparable analysis on both old and new genomes. The results showed that different genome assembles affected comparative analysis (Fig 6).

## Discussion and conclusion

Compared to other programs, Puzzle Hi-C uses Hi-C data to scaffold chromosome-level genomes while avoiding long-range interactions. Puzzle Hi-C uses a dynamic triangle window to calculate interaction densities. It dynamically changes the size of the triangle at both ends of the scaffold. Windows start small in initial iterations, which facilitates the assembling of smaller scaffolds, and excludes long-range interactions. While LACHHESIS [17], 3D DNA [25], and ALLHiC [27] use all interaction information between two scaffolds, such can result in errors in ordering due to their considering long-range interactions. As iterations increase in Puzzle Hi-C, the window size increases. This obtains superior chromosome clustering by selectively using all interaction information. It clusters scaffolds into chromosomes by sidestepping local interaction information only, unlike SALSA2 [26, 28] does. For the human genome, Puzzle Hi-C outperforms YaHS [30], ALLHiC [27], LACHESIS [17], 3D DNA [25], and SALSA2 [26, 28] in ordering and orientation in both simulated and real data, and with robust performance. The same result occurs upon applying Puzzle Hi-C to all other tested genomes. Further, Puzzle Hi-C outperforms other software when assembling the complex gayal genome, which has many long-range interactions. Whereas YaHS can produce fine-scale results without fine-tuning any parameters, Puzzle Hi-C needs the tuning of several parameters to obtain best results. Finally, the reassembled genome of the puffer fish reveals improvements when compared with the original assembly [34], and the results suggest that the genome-quality greatly impacts 3D genome analysis. Thus, accurate 3D genome analysis requires accurate chromosome-level genomes.

## Supporting information

S1 File (PDF)
