# Supplementary material for: Puzzle Hi-C: An accurate scaffolding software
Source: PLoS One. 2024 Jul 15;19(7):e0298564. doi: 10.1371/journal.pone.0298564 (PMC11249255; doi:10.1371/journal.pone.0298564)
Supplement: S1 File — (PDF) [file pone.0298564.s001.pdf]

Supplementary

S1 Text. Puzzle Hi-C pipeline

1.1 Hi-C assembly process design

To avoid existing problems is previous software, we employed a bottom-up iterative process to assemble chromosomes. We started from scaffolds and iteratively assembled them continuously into larger scaffolds. This process continued until the specified number of chromosomes was formed. This scheme avoided solving the traveling salesman problem (given a list of cities and the distances between each pair of cities, what is the shortest possible route that visits each city exactly once and returns to the origin city). We specified the length of scaffold endpoints to measure the relative distance between scaffolds, whose length *l* became larger as the number of iterations increased. The initial *l* by default was 30000 bp (*triangle size* * *bin-size*). The initial triangle and bin-size can be configured by -i and -s parameters. Relatively small values of *l* allowed for assembly based on local smoothness, thus avoiding the effect of distant interactions on assembly. As *l* increased, global information was used effectively for chromatin clustering. The specific process used mapping, ordering and orientation, and assembly in sequence. Ordering and orientation constituted the core steps.

1.2 Mapping

Sequence mapping used Juicer software with its bwa mem default parameters for mapping. Subsequently, Juicer filtered out PCR duplicates and retained one pairwise comparison only. After filtering out the duplicate and abnormal sequences, Juicer added enzyme cut-site information to the pairwise results for subsequent analysis. After obtaining the pairwise results, pairs with a MAPQ score < 30 were retained for subsequent analysis.

1.3 Ordering and Orientation

Puzzle Hi-C ordering and orientation uses an iterative algorithm. Processing stops when clustering equals the number of corresponding chromosomes.

Our software first constructs a graph during its ordering; nodes of the graph are scaffolds, and the number of Hi-C reads are take l-length at each end B and E of each scaffold for the pairs BB, BE, EB and EE. In comparison, LACHESIS and ALLHiC use global information to calculate weights; 3D DNA uses half of the data scaffolds to calculate the weights, and SALSA2 uses a fixed-length to calculate weights. Puzzle Hi-C is similar to SALSA2 in using a contig-end solution, but lengths increase during each iteration.

LACHESIS, ALLHiC and 3D DNA introduce bias when normalizing weights of different lengths due to the varying lengths of scaffolds. For example, when a scaffold becomes longer the programs decrease the weights of the scaffold with respect to other chromosomes. This bias substantially imparts the results when the lengths of scaffolds vary greatly. Therefore, in each iteration Puzzle Hi-C employs length *l* for its statistics, instead of using the percentage of scaffold lengths.

The number of Hi-C reads in the isosceles triangle interval of BB, BE, EB and EE connections is counted, and two scaffolds with the most Hi-C reads are joined.

$$N_{i,j} = \max\{N_{iB,jB}, N_{iB,jE}, N_{iE,jB}, N_{iE,jE}\}$$

Connectivity is record with an adjacency matrix.

$$O_{i,j} = \text{argmax}\{N_{iB,jB}, N_{iB,jE}, N_{iE,jB}, N_{iE,jE}\}$$

For each scaffold i, only the top 5 linked edges are considered. Next, a link-score is obtained for each pair as follows:

$$W_{topk} = \frac{N_{topk}}{\sum_{\mathrm{j}=1}^{5} N_{topj}}$$

Algorithm 1 Calculate weights and directions

Input: M is an $n * n * 4$ matrix, where $M_{i,j}$ holds the values corresponding to the four connections $N_{iB,jB}, N_{iB,jE}, N_{iE,jB}, N_{iE,jE}$.
Output: W is $n * n$ matrix storing the weights, L stores directions of any two scaffolds.

procedure CACULATE $W\&L$
    $scaffolds \leftarrow n; threshold \leftarrow m; i \leftarrow 0$
    while $i < scaffolds$ **do**
        $j \leftarrow i + 1$
        while $j < scaffolds$ **do**
            $W_{i,j} \leftarrow \max M_{i,j}$
            $L_{i,j} \leftarrow \arg\max M_{i,j}$
            $j \leftarrow j + 1$
        $i \leftarrow i + 1$
    $scaffolds \leftarrow n; i \leftarrow 0$
    while $i < scaffolds$ **do**
        $maxCount \leftarrow \max W_i$
        if $maxCount \leq threshold$ **then**
            $W_i \leftarrow 0$
        for j in argsort$W_i[n - 5 : n]$ **do**
            $sumCount \leftarrow sumCount + W_{i,j}$
        $sumCount \leftarrow \max sumCount, 1$
        $W_{i,i} \leftarrow sumCount$
        $W_i \leftarrow \frac{W_i}{sumCount}$
    return W, L

where scaffolds are the number n of all scaffolds involved in clustering and ordering, M is a matrix of $n*n*4$, which holds the four connections between all scaffolds, thus solving for the maximum value among the four connections to get unnormalized weights W and connection L. Finally, normalized weight W is calculated.

After determining W, each node is allowed to have at most two edges with the highest W. When W is less than the cutoff, the unreliable connection is eliminated. In the graph, all the paths with nodes > 1 are used to construct new scaffolds according to the path and connection direction for the next iteration. The length of l increases with the growth rate 1.4 to double the value every two iterations.

For the i round iteration, l_i calculated as follow:

$$l_i = \lfloor l_{i-1} \times 1.4 \rfloor$$

The iteration stopped only when the number of scaffolds equals or closely approaches the specified number of chromosomes.

Algorithm 2

```
Input: W
Output: G
procedure SOLVE G
    scaffolds ← n; cutoff ← m; i ← 0;
    G ← GRAPH; edges ← {};
    while i < scaffolds do
        twoPathIndex ← (argsort W_i)[n − 2 : n]
        for j in twoPathIndex do
            if i < j then
                edges.add((i, j))
            else
                edges.add((j, i))
        for edge in edges do
            x ← edge[0]
            y ← edge[1]
            score ← (W_{x,y} + W_{y,x}) / 2
            if score > cutoff then
                G.add_edge(x, y, weight = score)
        i ← i + 1
    return G
```

1.4 Error Correction in Puzzle Hi-C

Puzzle Hi-C has a function that can detect and correct scaffolding errors by mapping the distance of two reads and requiring that distances meet a specific threshold to minimize the impact of background noise. The distance $d_{i,j}$ satisfies the following inequality:

$$100 \text{ kb} < d_{i,j} < 500 \text{ kb}$$

Given position i, two blocks (blue and red rectangles in S4 Fig. a) satisfy the inequality in the Hi-C heat map (S3 Fig. a). The number of links in the blue rectangle is given as A_i and the number of links in green rectangle is B_i. The odds ratio $\lg(A_i/B_i)$ should approach 0. If an error exists, the value will deviate from zero (S4 Fig. b). We define an error score as follows:

$$E_i = \lg\,(A_{i-1}/B_{i-1}) - \lg(A_i/B_i)$$

The algorithm breaks the scaffold at the position *i* when E_i is larger than the cutoff. For example, in an insect genome YaHS slightly over-scaffolds, and Puzzle Hi-C

aggressively over-scaffolds without error correction (S5 Fig. a-b). Using the error correction function, Puzzle Hi-C generates a fine-scale result (S5 Fig. c). Notwithstanding, this function should not be used if Puzzle Hi-C generates a good result because this function will decrease the N50.

Puzzle Hi-C can also produce files for manual curation. A user can use “utils/agp2assembly.py” to generate an assembly file for manual curation. After curation, the user can use “utils/assembly2agp.py” to generate an agp file and use “utils/generate_fasta.py” to generate a fasta file.

Supplemental Figures

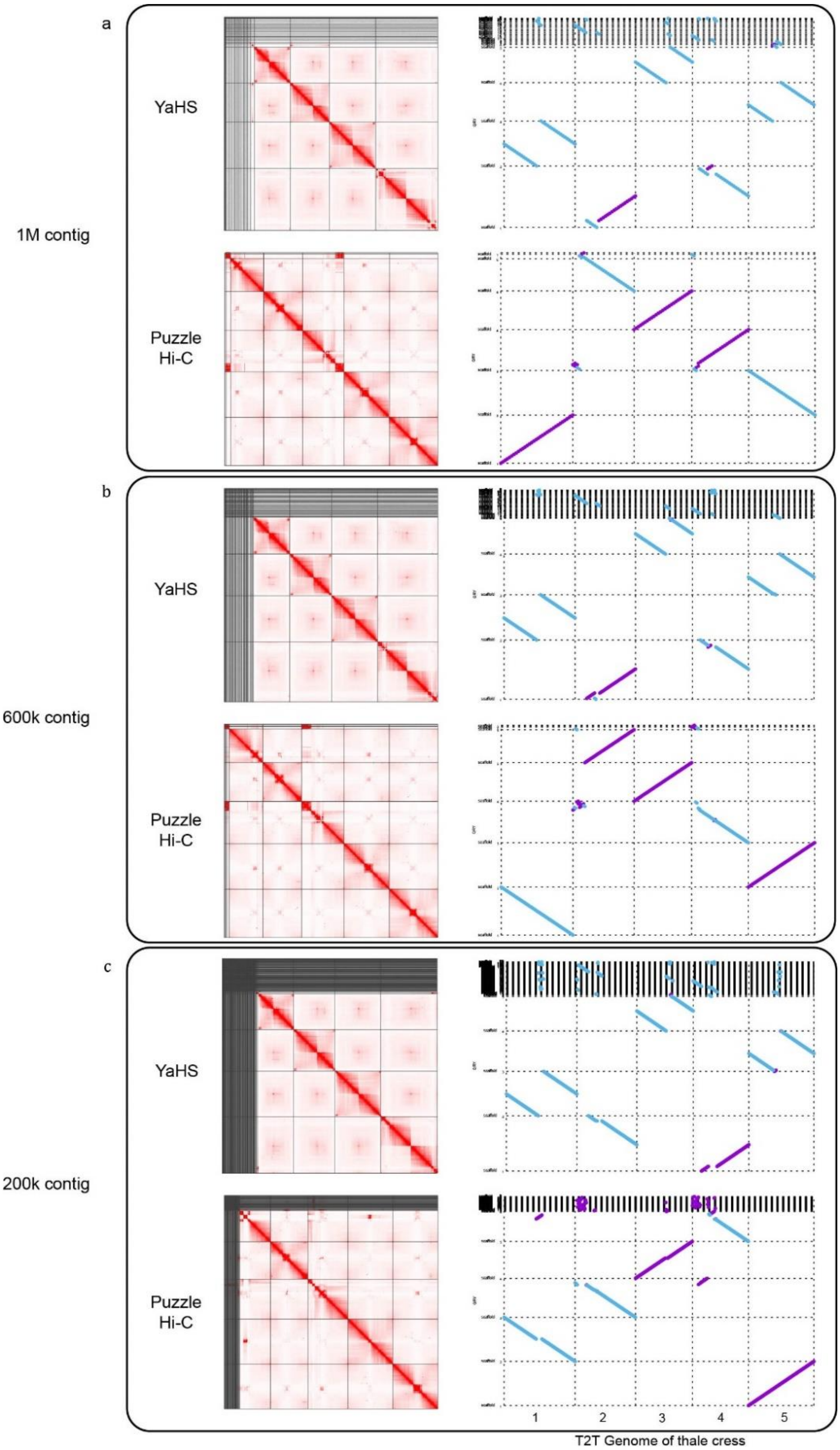

S1 Fig. Hi-C heat map and the synteny of genome assembled by YaHS and Puzzle Hi-C compared with The T2T genome of thale cress. a-c Different contig lengths used for scaffolding. All results are raw output without manual curation. The Hi-C heat maps are flipped to correspond with the synteny maps.

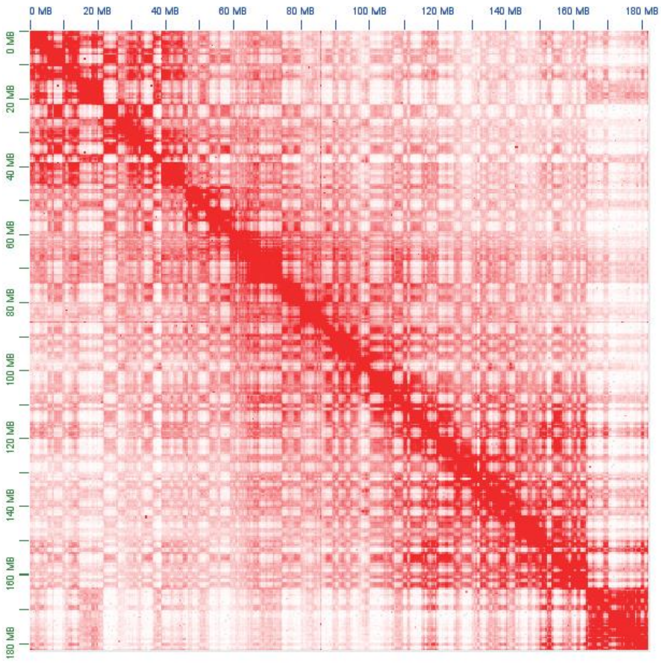

S2 Fig. Hi-C heat map of gayal chr02 showing chromosomal enrichment in long-distance interactions.

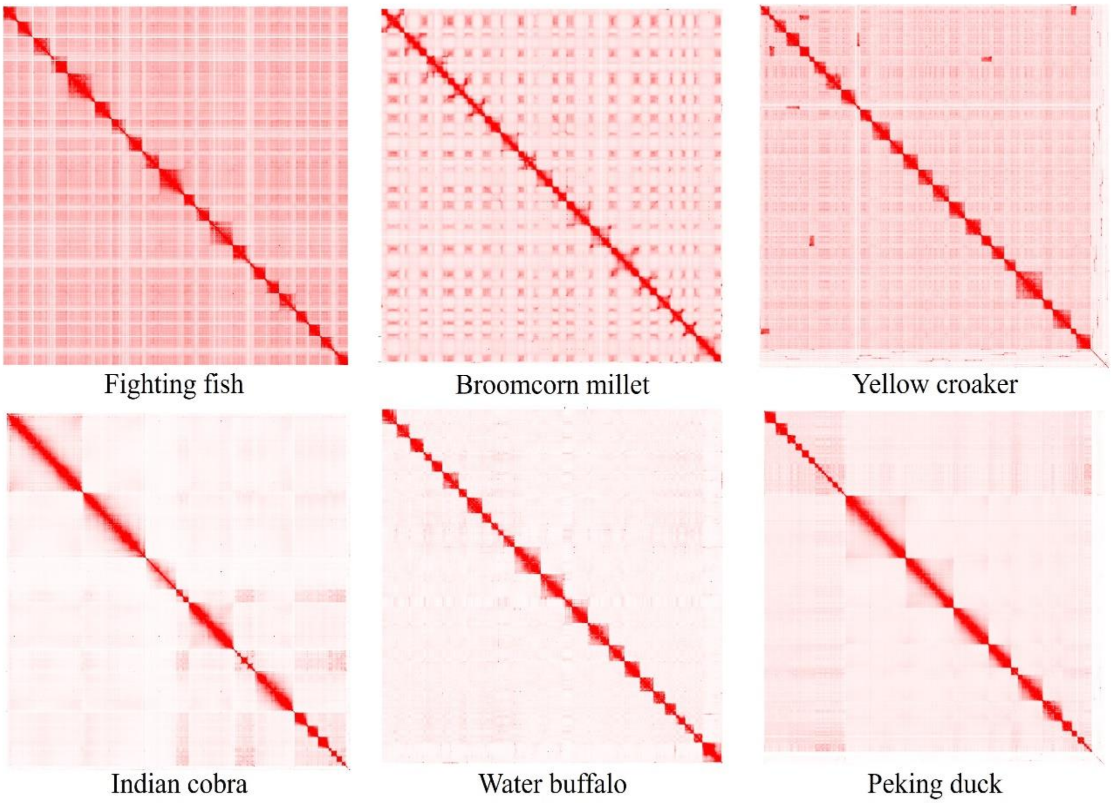

S3 Fig. Hi-C heat maps of assemblies constructed from Puzzle Hi-C from raw output.

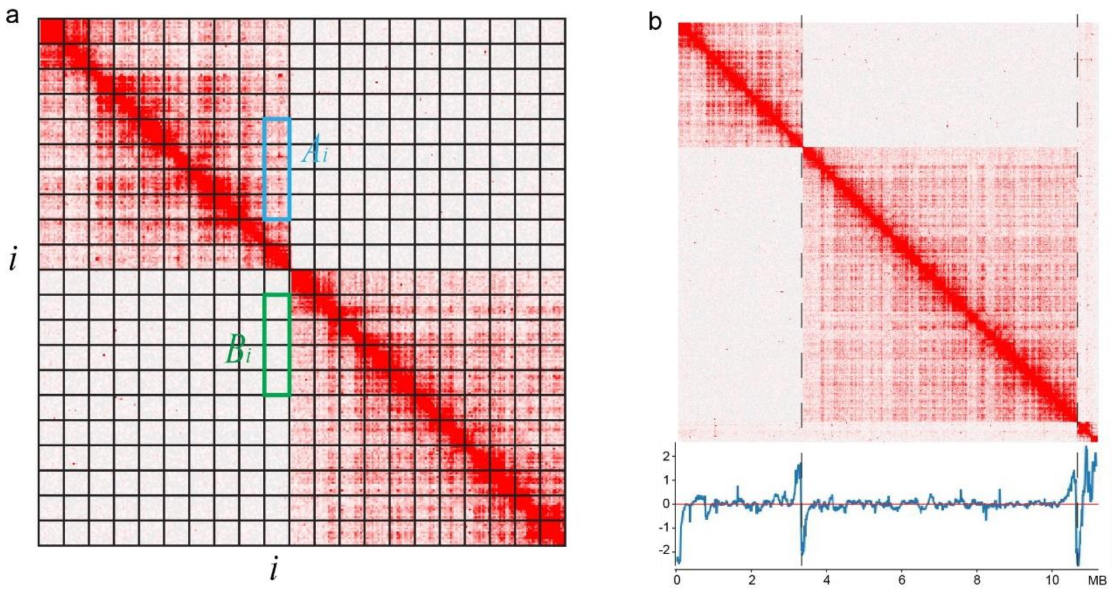

S4 Fig. Error detection method of Puzzle Hi-C. a, Links in blue and green rectangles are used for calculating error scores. **b**, Error detection with the visualized odds ratio. Black dashes indicate the break point and horizontal red line is the theoretical value of the error score.

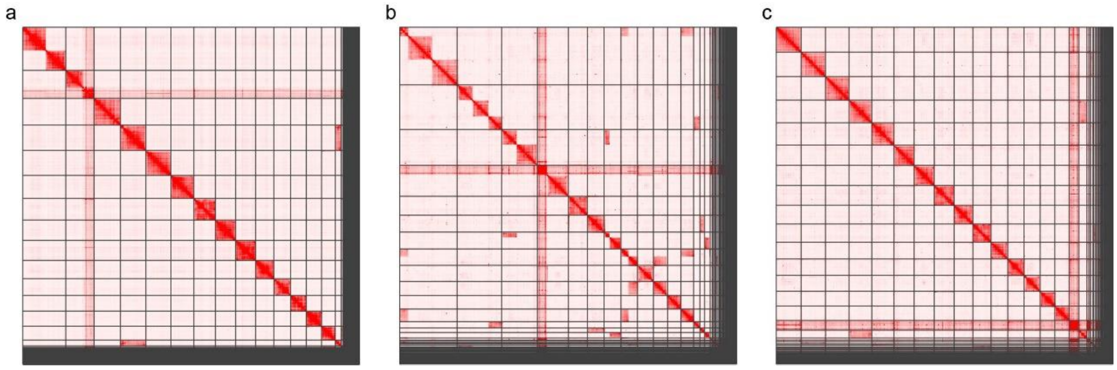

S5 Fig. Hi-C heat maps of flower thrips genome assembled by YaHS and Puzzle Hi-C. a, Genome assembled by YaHS. **b**, Genome assembled by Puzzle Hi-C without error correction. **c**, Genome assembled by Puzzle Hi-C with error correction.

Supplemental Tables

S1 Table. SRA data used in this study

Organism	BioProject	Run Accession No.	Reference
Human	PRJNA268125	SRR1658570	GCA_000001405.29; GCA_001013985.1
Thale cress	PRJCA005809 (NGDC)	CRR302669	GCA_028009825.2
Gayal	PRJNA612712	SRR23944069	PRJNA612712
Puffer fish	PRJNA508537	SRR8285225	GCA_004026145.2
Broomcorn millet	PRJNA429322	SRR7164797; SRR7164798	GCA_002895445.2
Indian cobra	PRJNA527614	SRR10420848; SRR10420847	GCA_009733165.1
Peking duck	PRJNA636121	SRR11910010	GCA_015476345.1
Water buffalo	PRJNA437177	SRR7284794; SRR7284796; SRR7284798	GCA_003121395.1
Large yellow Croaker	PRJNA505927	SRR8208301	GCA_003845795.1
Fighting fish	PRJNA416843	SRR6251367	GCA_013403625.1
Flower thrips	PRJNA1027977	SRR2638472849	GCA_033675135.1

S2 Table. LACHESIS scaffolding results

	200 kb	600 kb	1 Mb
Total sizes (Gb)	2.77	2.77	2.77
Total scaffolds	102	37	33
N50 (Mb)	135.35	135.11	135.10
L50	9	9	9
N90 (Mb)	79.81	68.83	77.45
L90	19	19	19

S3 Table. SALSA2 scaffolding results

	200 kb	600 kb	1 Mb
Total sizes (Gb)	2.78	2.77	2.77
Total scaffolds	1193	598	538
N50 (Mb)	8.62	9.30	9.98
L50	99	84	89
N90 (Mb)	1.20	2.40	2.84
L90	364	297	295

S4 Table. 3D DNA scaffolding results

	200 kb	600 kb	1 Mb
Total sizes (Gb)	2.77	2.77	2.77
Total scaffolds	368	119	150
N50 (Mb)	131.14	121.22	40.00
L50	9	10	24
N90 (Mb)	32.02	33.01	16.00
L90	24	24	61

S5 Table. ALLHiC scaffolding results

	200 kb	600 kb	1 Mb
Total sizes (Gb)	2.77	2.77	2.77
Total scaffolds	22	22	22
N50 (Mb)	135.75	135.11	135.10
L50	9	9	9
N90 (Mb)	80.21	68.83	67.45
L90	19	19	19

S6 Table. YaHS scaffolding results

	200 kb	600 kb	1 Mb
Total sizes (Gb)	2.77	2.77	2.77
Total scaffolds	1028	246	408
N50 (Mb)	140.03	142.66	131.70
L50	8	8	8
N90 (Mb)	59.18	76.09	59.27
L90	19	18	19

S7 Table. Puzzle Hi-C scaffolding results

	200 kb	600 kb	1 Mb
Total sizes (Gb)	2.77	2.77	2.77
Total scaffolds	703	191	109
N50 (Mb)	128.46	130.91	154.36
L50	8	8	8
N90 (Mb)	44.82	55.63	56.62
L90	20	18	18

S8 Table. Summary of errors generated by different software with 25 resamples

Contig Length	Software	Relocations	Translocations	Inversions	Total errors
**200k**	3D DNA	930	265	2898	4093
	ALLHiC	1407	207	2053	3667
	LACHESIS	963	118	919	2000
	Puzzle Hi-C	82	12	260	354
	SALSA	877	61	2044	2982
	YaHS	69	0	311	380
**400k**	3D DNA	368	100	1868	2336
	ALLHiC	570	81	768	1419
	LACHESIS	474	54	556	1084
	Puzzle Hi-C	55	12	155	222
	SALSA	630	38	1303	1971
	YaHS	34	1	133	168
**600k**	3D DNA	229	87	1114	1430
	ALLHiC	381	50	446	877
	LACHESIS	286	52	374	712
	Puzzle Hi-C	91	31	114	236
	SALSA	1099	30	1516	2645
	YaHS	4	1	56	61
**800k**	3D DNA	200	65	956	1221
	ALLHiC	258	47	324	629
	LACHESIS	187	46	291	524
	Puzzle Hi-C	48	36	114	198
	SALSA	1570	32	1374	2976
	YaHS	75	6	112	193
**1M**	3D DNA	146	52	729	927
	ALLHiC	192	37	266	495
	LACHESIS	194	26	181	401
	Puzzle Hi-C	33	37	74	144
	SALSA	539	17	1094	1650
	YaHS	73	5	76	154

S9 Table. Number of errors generated by YaHS and Puzzle Hi-C with the T2T genome of thale cress

Contig Length	Error type	YaHS	Puzzle Hi-C
**200k**	Relocations	4	1
	Translocations	1	7
	Inversions	2	6
**600k**	Relocations	4	1
	Translocations	1	6
	Inversions	5	6
**1M**	Relocations	4	1
	Translocations	1	5
	Inversions	3	0
